# Effect of Common Staining Beverages on the Color Stability of Additively and Subtractively Manufactured Provisional Materials

**DOI:** 10.3390/dj14030164

**Published:** 2026-03-12

**Authors:** Alberto Ferreiroa, Michelle Lissette Ortiz-Soto, Jaime Orejas, Irene García-Martínez, Laura Godoy-Ruiz

**Affiliations:** 1 Department of Restorative Dentistry and Prosthodontics, Faculty of Dentistry, Complutense University of Madrid, 28040 Madrid, Spain; 2 Department of Odontology, Faculty of Health Sciences, European University of Madrid, 28670 Villaviciosa de Odón, Spain; michelleortizsoto97@gmail.com (M.L.O.-S.); jaime.orejas@universidadeuropea.es (J.O.); icgmdental@gmail.com (I.G.-M.); laura.godoy@universidadeuropea.es (L.G.-R.)

**Keywords:** additive manufacturing, color stability, dental materials, spectrophotometry, subtractive manufacturing, three-dimensional printing

## Abstract

**Background/Objectives**: The increasing use of digital manufacturing techniques in prosthodontics has raised concerns regarding the long-term esthetic performance of provisional restorations. This in vitro study aimed to compare the color stability of provisional restorations fabricated by additive (3D-printed) and subtractive (milled) manufacturing techniques after immersion in common staining beverages. **Methods**: Eighty polymethyl methacrylate (PMMA)-based specimens (16 × 1 mm) were fabricated and divided into two groups: additive (n = 40) and subtractive (n = 40). Each group was immersed in coffee, red wine, green tea, or cola for 60 and 120 h at 37 °C. Color measurements were recorded using a spectrophotometer in the CIE LCh* system, and color differences (ΔE_00_) were calculated using the CIEDE2000 formula. Data were analyzed using repeated-measures analysis of variance (ANOVA) and Tukey post hoc tests (α = 0.05). **Results**: Additively manufactured specimens showed significantly higher color change (ΔE_00_) values than subtractively milled specimens across all immersion media and time intervals (*p* < 0.05). Red wine and coffee caused the greatest discoloration, whereas cola produced the least effect. **Conclusions**: Within the limitations of this study, additive manufacturing resulted in lower color stability than subtractive fabrication. Subtractive PMMA materials exhibited superior optical behavior, suggesting their suitability for long-term provisionalization in esthetically demanding clinical situations.

## 1. Introduction

Provisional restorations are essential components of fixed prosthodontic treatment, providing interim function, esthetics, and biological protection during the interval between tooth preparation and placement of the definitive prosthesis. With growing patient expectations and extended treatment durations, the optical performance of these interim restorations, particularly their color stability, has become clinically significant. The visual appearance of provisional materials is not only important for patient satisfaction during treatment but also serves as a diagnostic reference for shade selection and soft tissue management in definitive prosthetic rehabilitation [1].

Discoloration of provisional materials can result from a variety of intrinsic and extrinsic factors [2]. Intrinsic factors include the chemical composition and polymer structure of the material, the degree of polymer conversion, and water sorption capacity. Surface texture and finishing also play key roles in susceptibility to staining. Extrinsic factors such as oral hygiene, smoking, and dietary habits, particularly the consumption of chromogenic beverages such as coffee, tea, red wine, and cola-based drinks, are known contributors to color degradation [3]. In clinical situations where provisional restorations must remain in place for extended periods, such as in complex full-mouth rehabilitations or staged implant therapies, maintaining color stability becomes a functional and esthetic necessity [4].

Color change in provisional materials may occur through different mechanisms depending on the interaction between the material and chromogenic agents. Extrinsic discoloration is mainly associated with the adsorption and absorption of pigments from staining beverages, whereas intrinsic color changes may be related to the chemical characteristics of the polymer matrix. Components such as residual monomers, pigments, plasticizers, and the degree of polymerization can influence the susceptibility of resin-based materials to staining, particularly during prolonged exposure to chromogenic solutions.

In recent years, advances in digital dentistry, especially in computer-aided manufacturing workflows, have enabled the fabrication of provisional restorations with improved precision, reduced human error, and greater efficiency [5]. These techniques comprise both subtractive and additive manufacturing approaches: the former involves milling restorations from pre-polymerized polymethyl methacrylate (PMMA) blocks, while the latter constructs them layer by layer using 3D printing technologies [6]. While subtractive manufacturing benefits from the industrial fabrication of dense, highly polymerized materials with superior mechanical and optical stability, additive manufacturing provides workflow flexibility, cost efficiency, and customization potential. However, the layered structure and photopolymer chemistry of additive resins may result in increased porosity, a lower degree of polymer conversion, and surface heterogeneity, factors that could compromise long-term color stability [7].

The increasing integration of digital workflows in daily clinical practice has led to the widespread use of both CAD/CAM milling and chairside 3D printing for provisional restorations. While additive manufacturing offers advantages in terms of speed and cost-effectiveness, concerns remain regarding the long-term esthetic performance of 3D-printed provisional materials, particularly in cases requiring extended intraoral service.

Although several studies have evaluated the mechanical properties of digitally fabricated provisional materials, relatively few have addressed their color stability under simulated oral conditions [8,9]. Existing studies often focus on a single manufacturing method or lack standardized exposure protocols. Consequently, evidence directly comparing additive and subtractive manufactured provisional materials remains limited, and clinicians lack objective data to guide material selection for esthetically demanding cases [10,11]. Moreover, limited evidence is available comparing additively and subtractively manufactured provisional materials using perceptually uniform color difference formulas under standardized staining conditions.

This in vitro study was designed to assess the color stability of PMMA-based provisional restorations fabricated using additive (3D-printed) and subtractive (milled) manufacturing techniques. Color changes were quantified using the CIEDE2000 (ΔE_00_) formula to provide a clinically relevant assessment of perceptible color differences. The null hypothesis of this study was that there would be no significant difference in the color stability of PMMA-based provisional materials fabricated using additive or subtractive manufacturing methods.

## 2. Materials and Methods

**Figure 1:**
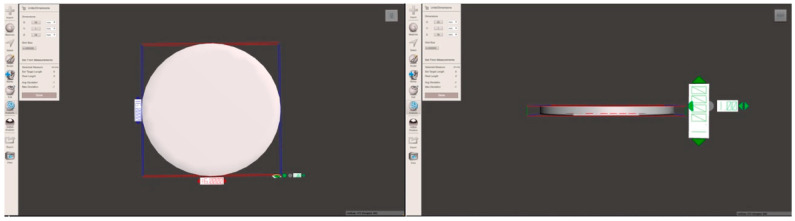
CAD design of the disc used in the study following the ISO norm 6872.

A total of 80 disc-shaped specimens (16 mm diameter × 1 mm thickness) were designed following ISO 6872:2015 guidelines [12] and prepared using computer-aided design (CAD) software (Meshmixer version 3.5, Autodesk Inc., San Rafael, CA, USA) (Figure 1).

Forty specimens were fabricated using an additive manufacturing process with a 3D printer (Photon S, Anycubic, Shenzhen, China) and a light-cured biocompatible resin (GC Temp Print Light, GC Corporation, Tokyo, Japan), specifically formulated for intraoral use in provisional restorations. The specimens were printed in 50 μm layers and post-cured in a UV polymerization unit according to the manufacturer’s instructions.

The remaining forty specimens were fabricated by subtractive manufacturing using a 5-axis dry milling machine (DWX-52D, Roland DG Corporation, Shizuoka, Japan). These discs were milled from industrially polymerized PMMA blocks (Huge PMMA Block B1, Aidite, Qinhuangdao, China), ensuring a high material density and minimal residual monomer content.

All specimens underwent standardized mechanical polishing with a universal polishing paste (Ivoclar Vivadent, Schaan, Liechtenstein) applied with a soft-bristle rotary brush at low speed for 20 s per surface. This was followed by ultrasonic cleaning in distilled water for 10 min to eliminate any residual particles or contaminants.

The 80 specimens were randomly allocated into two main groups according to the fabrication method: Group A (additive manufacturing; n = 40) and Group B (subtractive manufacturing; n = 40). Each group was further divided into four subgroups (n = 10 per subgroup) according to the immersion medium:

- Subgroup 1: coffee (Nescafé Classic, prepared with 2 g of powder dissolved in 200 mL of distilled water);
- Subgroup 2: cola-based beverage (Coca-Cola™, The Coca-Cola Company, Atlanta, GA, USA);
- Subgroup 3: red wine (Cabernet Sauvignon, 12% alcohol by volume);
- Subgroup 4: green tea (Lipton, Unilever, London, UK, prepared with a 2 g sachet infused in 200 mL of distilled water).

All beverages were freshly prepared daily and maintained at room temperature (22 ± 1 °C) under light-protected conditions. Each specimen was individually placed in a sealed, labeled plastic container filled with 50 mL of the corresponding chromogenic solution and stored without agitation. Solutions were renewed every 24 h to maintain consistent pH and pigment concentration. Specimens were completely immersed in the staining solutions throughout the experimental period.

Color measurements were performed at three time points: baseline (T_0_), after 60 h of immersion (T_1_), and after 120 h (T_2_). These immersion periods were selected to simulate different durations of exposure to chromogenic beverages during clinical use of provisional restorations. At each time point, specimens were removed from the solution, rinsed with distilled water, gently air-dried, and immediately subjected to color analysis.

**Figure 2:**
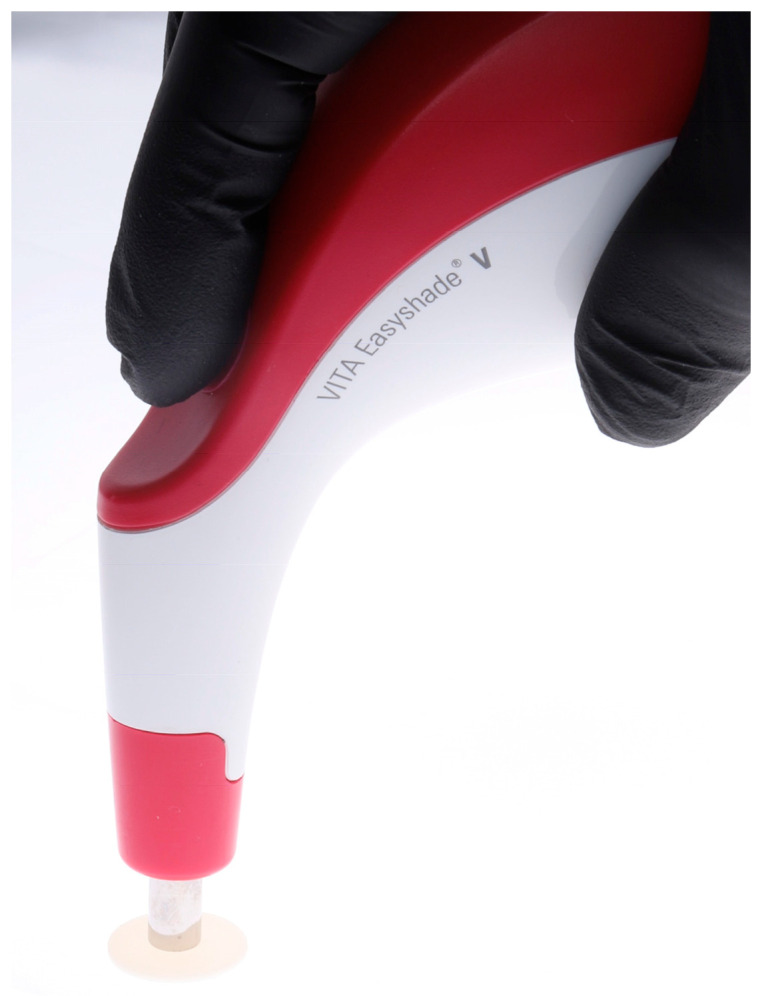
Spectrophotometer used to evaluate the specimens.

Color evaluation was conducted using a calibrated spectrophotometer (VITA Easyshade V, VITA Zahnfabrik, Bad Säckingen, Germany), which uses LED illumination and photodiode array detection to record the reflected light spectrum. Each measurement was performed in triplicate per specimen and averaged to ensure reproducibility (Figure 2). The spectrophotometer was calibrated prior to each measurement session following the manufacturer’s recommendations.

**Figure 3:**
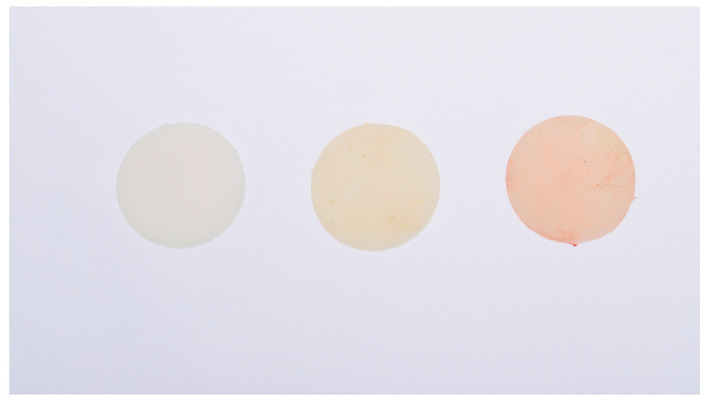
Representative PMMA disc specimens fabricated by additive manufacturing at baseline (T_0_), after 60 h (T_1_), and 120 h (T_2_) of immersion in red wine, illustrating the progressive discoloration observed under standardized in vitro conditions.

The CIE LCh* color space was used for color characterization, where L* denotes lightness (ranging from 0 for black to 100 for white), C* represents chroma or color saturation, and h* corresponds to hue angle, indicating the color type in degrees (Figure 3).

Color differences (ΔE_00_) were calculated at each time point using the CIEDE2000 formula, which provides improved perceptual correlation and accounts for visual non-uniformities.

Statistical analysis was performed using SPSS Statistics 27.0 (IBM Corp., Armonk, NY, USA). Data normality was assessed with the Shapiro–Wilk test, and homogeneity of variances was verified using Levene’s test. Repeated-measures analysis of variance (ANOVA) was applied to evaluate the effects of fabrication technique, immersion medium, and time on color change values. When significant effects were identified, Tukey’s honestly significant difference (HSD) test was used for post hoc comparisons. The level of significance was set at α = 0.05, and 95% confidence intervals and effect sizes (η^2^) were reported where applicable.

## 3. Results

Regarding color changes according to the manufacturing technique, at both T_1_ and T_2_, specimens fabricated using the additive technique exhibited greater changes in all color parameters compared to those manufactured using the subtractive technique across all chromogenic solutions evaluated.

In terms of lightness (ΔL*), at T_2_, the mean value for specimens in Group A immersed in coffee was −16.62 ± 5.19, indicating a significant decrease in brightness. In contrast, specimens from Group B in the same solution showed a mean ΔL* of −0.26 ± 0.93, reflecting only minimal change. Comparable trends were observed for the other staining solutions (Table 1).

For chroma (ΔC*), Group A exhibited more pronounced reductions, particularly in the red wine and coffee subgroups, with mean values of −14.34 ± 2.20 and −9.92 ± 3.39, respectively. In comparison, Group B showed smaller shifts, such as a ΔC* of −5.26 ± 0.59 in the red wine subgroup (Table 2).

The hue angle variation (Δh*) was consistently higher in Group A compared with Group B. At T_1_, the most notable hue changes were observed in specimens immersed in coffee and red wine within Group A, whereas those in Group B remained relatively stable. By T_2_, green tea and cola beverages produced the most pronounced hue shifts in Group A, with values of −14.60 ± 5.84 and −14.34 ± 2.20, respectively (Table 3).

Repeated-measures ANOVA revealed statistically significant differences in color change based on manufacturing technique, immersion time, and type of staining solution (*p* < 0.05). For instance, the difference in ΔL* between Group A and Group B at T_2_ was statistically significant for both red wine (*p* = 0.001) and green tea (*p* < 0.001), with higher values recorded in the additive manufacturing group (Table 4 and Table 5).

Post hoc analysis using the Tukey HSD test identified the greatest intergroup differences in specimens immersed in coffee and cola beverages (*p* = 0.016), particularly in the ΔL* and ΔC* parameters (Table 6).

## 4. Discussion

The findings of this in vitro study reject the null hypothesis, as significant differences in color stability were observed between provisional restorations fabricated using additive and subtractive manufacturing techniques. Specimens produced by additive manufacturing demonstrated significantly greater discoloration across all evaluated parameters (ΔL*, ΔC*, and Δh*) at both 60 and 120 h of immersion when compared with their subtractive counterparts. These results underscore the substantial impact of the fabrication method on the optical stability of PMMA-based provisional materials, particularly under exposure to chromogenic agents in simulated intraoral conditions relevant to digital dentistry workflows.

These observations are consistent with previous studies reporting increased susceptibility to staining among 3D-printed resins. Song et al. [8] reported significant color alterations in 3D-printed provisional restorations, particularly after 8 weeks of immersion in coffee and tea. In contrast, milled PMMA blocks initially demonstrated better color stability but experienced a significant increase in ΔE_00_ values after prolonged exposure. This pattern aligns with the current findings and reinforces the long-term optical superiority of pre-polymerized milled materials for applications requiring extended esthetic stability [13].

The intrinsic characteristics of additive manufacturing, particularly its layer-by-layer polymerization process under atmospheric pressure, may explain the observed outcomes. This technique is associated with lower degrees of polymer conversion and increased residual porosity, which facilitates the absorption and retention of chromogenic agents. Previous studies have similarly demonstrated that specimens fabricated through additive methods exhibit greater staining susceptibility and reduced overall color stability compared with milled PMMA blocks [14].

The staining hierarchy observed in this study is consistent with previous literature, with coffee and red wine inducing the most pronounced discoloration. Song et al. [8] emphasized that chromogenic beverages rich in polyphenols, such as coffee and tea, exert a cumulative staining effect, especially on materials with higher surface reactivity and water sorption. Interestingly, the carbonated cola beverage induced the least color change, a finding that may be attributed to its relatively low chromogen content despite its acidity and its limited pigment adsorption capacity.

When the present results are interpreted in light of established perceptibility and acceptability thresholds for color differences, their clinical relevance becomes more evident. Previous studies have suggested that ΔE_00_ values exceeding approximately 1.8 are perceptible to the human eye, while values above 3.3 are considered clinically unacceptable. In the present study, additively manufactured provisional materials frequently exceeded the perceptibility threshold and, in some staining conditions and time intervals, also surpassed the acceptability threshold. In contrast, subtractively manufactured PMMA materials generally remained below or close to the clinically unacceptable threshold, particularly at shorter immersion times. These findings indicate that the color changes observed in additively manufactured specimens may be clinically noticeable and potentially esthetically compromising during prolonged provisionalization.

The selection of 60 and 120 h as immersion time points was strategically intended to simulate two clinically relevant exposure periods: a short-term phase corresponding to brief provisional use (2–3 days) and a longer-term phase (>5 days) corresponding to complex or staged prosthetic treatments. This temporal design allows for the assessment of both immediate and progressive color changes, reflecting real-world intraoral conditions where provisional restorations may be exposed to chromogenic agents for varying durations. An in vitro study by Al-Alkhali et al. [14] demonstrated that the duration of immersion significantly affected the discoloration of both digitally and manually fabricated provisional materials, with greater color changes observed as exposure time increased. These findings underscore the importance of including multiple time intervals in color stability studies to capture the dynamic progression of staining effects. By evaluating color changes at 60 and 120 h, the current study provides a clinically meaningful perspective on the temporal behavior of provisional materials in esthetically demanding scenarios. Although 120 h does not directly correspond to a continuous clinical exposure period, it represents an accelerated in vitro model commonly used to simulate cumulative staining effects during prolonged provisionalization in complex restorative treatments.

From a clinical standpoint, the present findings are particularly relevant for practices increasingly relying on digital workflows and chairside manufacturing. While additively manufactured provisional restorations offer advantages in terms of speed, cost-efficiency, and workflow simplification, their reduced long-term color stability should be carefully considered when prolonged provisionalization is anticipated.

Clinically, these data suggest that although additive manufacturing is efficient for short-term restorations, its limitations in long-term esthetic durability remain a concern. The superior performance of subtractive PMMA blocks, attributable to their dense, homogeneous microstructure, makes them a preferable option for esthetically sensitive clinical scenarios [15,16]. Previous studies have reported that esthetic perception and shade evaluation may differ between clinicians and patients, reinforcing the role of provisional restorations not only as interim solutions but also as esthetic references during definitive prosthetic planning [17].

Nevertheless, color stability is a multifactorial phenomenon influenced by parameters such as surface finish, polishing protocol, and intraoral environmental conditions [18]. Water sorption and solubility have been shown to exacerbate discoloration over time, although all materials evaluated in this study remained within the acceptable limits established by ISO standards [19]. While it is not feasible to standardize all contributing variables, clinical factors such as dietary habits, oral hygiene, and salivary composition may further impact the clinical performance of provisional restorations.

A limitation of this study lies in its in vitro design, which may not fully replicate the complexity of the intraoral environment. Future research should incorporate additional dynamic elements such as thermocycling, simulated brushing, and extended aging protocols to assess real-world performance.

In addition, although standardized polishing protocols were applied to all specimens, quantitative surface roughness was not assessed. Surface topography may influence staining behavior and should be considered in future studies correlating surface roughness parameters with color stability outcomes [20].

From a methodological perspective, only one specimen thickness and a single printing layer thickness were evaluated, which may limit the generalizability of the findings to other clinical configurations. Moreover, the use of a single material per manufacturing technique does not account for potential variability among different commercially available provisional materials. Finally, although standardized polishing and immersion protocols were applied, mechanical wear and other intraoral factors were not simulated and may influence color stability under clinical conditions.

Moreover, ongoing material innovations and surface modification strategies, especially for 3D-printable resins, as well as improvements in resin formulation and post-curing protocols, hold promise for improving clinical longevity.

## 5. Conclusions

Within the limitations of this in vitro study, the color stability of provisional restorations was significantly influenced by the manufacturing technique. Additively fabricated specimens showed greater discoloration than subtractively milled ones across all staining solutions, with coffee and red wine producing the most pronounced color changes, while cola beverages exhibited the least effect.

Subtractive materials demonstrated superior optical stability, likely due to their higher polymer conversion and more homogeneous structure, making them more suitable for long-term provisionalization in esthetic areas within digital prosthodontic workflows.

Clinically, additive manufacturing remains a convenient option for short-term restorations, but its esthetic limitations under prolonged exposure should be carefully considered, particularly in digitally driven chairside workflows. Further in vivo studies are recommended to support the development of improved 3D-printed resins with enhanced resistance to staining.

## Figures and Tables

**Table 1:** Mean (±SD) changes in lightness (ΔL*) of additively and subtractively manufactured provisional materials after immersion in different staining beverages.

Beverage	Additive T1 (60 h) (Mean ± SD)	Additive T2 (120 h) (Mean ± SD)	Subtractive T1 (60 h) (Mean ± SD)	Subtractive T2 (120 h) (Mean ± SD)
Coffee	−8.21 ± 2.15	−16.62 ± 5.19	−0.15 ± 0.74	−0.26 ± 0.93
Cola	−4.33 ± 1.20	−9.66 ± 1.91	−0.10 ± 0.38	−0.21 ± 0.47
Red Wine	−2.44 ± 0.88	−5.62 ± 1.42	−0.21 ± 0.29	−0.41 ± 0.56
Green Tea	−1.02 ± 0.65	−2.25 ± 1.00	−0.18 ± 0.35	−0.38 ± 0.44

SD: standard deviation. ΔL*: lightness change.

**Table 2:** Mean (±SD) changes in chroma (ΔC*) of additively and subtractively manufactured provisional materials after immersion in different staining beverages.

Beverage	Additive T1 (60 h) (Mean ± SD)	Additive T2 (120 h) (Mean ± SD)	Subtractive T1 (60 h) (Mean ± SD)	Subtractive T2 (120 h) (Mean ± SD)
Coffee	7.32 ± 1.45	−0.92 ± 3.39	−2.04 ± 0.85	−2.48 ± 1.13
Cola	−5.98 ± 1.33	−14.34 ± 2.20	−2.63 ± 0.61	−5.26 ± 0.59
Red Wine	−3.42 ± 1.12	−9.22 ± 2.01	−2.85 ± 0.74	−5.74 ± 0.82
Green Tea	−2.15 ± 1.04	−14.60 ± 5.84	−2.71 ± 0.96	−5.96 ± 1.03

SD: standard deviation. ΔC*: chroma change.

**Table 3:** Mean (±SD) changes in hue angle (Δh*) of additively and subtractively manufactured provisional materials after immersion in different staining beverages.

Beverage	Additive T1 (60 h) (Mean ± SD)	Additive T2 (120 h) (Mean ± SD)	Subtractive T1 (60 h) (Mean ± SD)	Subtractive T2 (120 h) (Mean ± SD)
Coffee	−4.33 ± 1.55	−5.25 ± 2.34	−0.96 ± 0.68	−1.24 ± 0.71
Cola	−6.92 ± 1.82	−14.34 ± 2.20	−1.17 ± 0.53	−2.00 ± 0.68
Red Wine	−4.10 ± 1.27	−8.72 ± 1.90	−1.33 ± 0.61	−2.60 ± 0.77
Green Tea	−5.90 ± 1.40	−14.60 ± 5.84	−1.09 ± 0.50	−2.17 ± 0.66

SD: standard deviation. Δh*: hue angle change.

**Table 4:** Repeated-measures ANOVA results for color changes of additively manufactured provisional materials according to staining beverage and immersion time.

Parameter	Time Point	F-Value	*p*-Value
ΔL*	T_1_	3.058	0.059
ΔL*	T_2_	7.658	0.002
ΔC*	T_1_	8.798	0.001
ΔC*	T_2_	22.672	<0.001
Δh*	T_1_	8.871	0.001
Δh*	T_2_	25.988	<0.001

ANOVA: analysis of variance.

**Table 5:** Repeated-measures ANOVA results for color changes of subtractively manufactured provisional materials according to staining beverage and immersion time.

Parameter	Time Point	F-Value	*p*-Value
ΔL*	T_1_	614	0.616
ΔL*	T_2_	1.906	0.169
ΔC*	T_1_	84.015	<0.001
ΔC*	T_2_	27.311	<0.001
Δh*	T_1_	916	0.456
Δh*	T_2_	3.329	0.046

ANOVA: analysis of variance.

**Table 6:** Tukey post hoc intergroup comparisons between additively and subtractively manufactured provisional materials after immersion in different staining beverages.

Group	Beverages	ΔL*	ΔC*	Δh*
Additive manufacturing (Mean ± SD)	Coffee vs. cola	*p* < 0.001	*p* < 0.001	*p* < 0.001
	Coffee vs. red wine	*p* = 0.002	*p* = 0.011	*p* = 0.017
	Coffee vs. green tea	*p* = 0.009	*p* = 0.005	*p* = 0.020
	Cola vs. red wine	*p* = 0.038	*p* = 0.024	*p* = 0.001
	Cola vs. green tea	*p* = 0.042	*p* = 0.007	*p* = 0.011
	Red wine vs. green tea	*p* = 0.065	*p* = 0.045	*p* = 0.039
Subtractive manufacturing (Mean ± SD)	Coffee vs. cola	*p* = 0.552	*p* = 0.379	*p* = 0.600
	Coffee vs. red wine	*p* = 0.411	*p* = 0.465	*p* = 0.478
	Coffee vs. green tea	*p* = 0.398	*p* = 0.387	*p* = 0.443
	Cola vs. red wine	*p* = 0.733	*p* = 0.694	*p* = 0.745
	Cola vs. green tea	*p* = 0.612	*p* = 0.710	*p* = 0.702
	Red wine vs. green tea	*p* = 0.551	*p* = 0.683	*p* = 0.654

## Data Availability

The data presented in this study are available from the corresponding author upon reasonable request.
